# HIF-1α stimulates aromatase expression driven by prostaglandin E_2 _in breast adipose stroma

**DOI:** 10.1186/bcr3410

**Published:** 2013-04-08

**Authors:** Nirukshi U Samarajeewa, Fangyuan Yang, Maria M Docanto, Minako Sakurai, Keely M McNamara, Hironobu Sasano, Stephen B Fox, Evan R Simpson, Kristy A Brown

**Affiliations:** 1 Prince Henry's Institute, Block E Level 4, Monash Medical Centre, 246 Clayton Rd, Clayton, Melbourne, VIC 3168, Australia; 2 Department of Physiology, Monash University, Clayton, Melbourne, VIC 3168, Australia; 3 Department of Pathology, Tohoku University, 2-1 Seiryo-machi, Aoba-ku, Sendai, Miyagi 980-8575, Japan; 4 Department of Pathology, Peter MacCallum Cancer Centre, Melbourne, VIC 8006, Australia; 5 Department of Pathology, Melbourne University, Parkville, VIC 3010, Australia; 6 Department of Biochemistry and Molecular Biology, Monash University, Clayton, Melbourne, VIC 3168, Australia

## Abstract

**Introduction:**

The majority of postmenopausal breast cancers are estrogen-dependent. Tumor-derived factors, such as prostaglandin E_2 _(PGE_2_), stimulate CREB1 binding to cAMP response elements (CREs) on aromatase promoter II (PII), leading to the increased expression of aromatase and biosynthesis of estrogens within human breast adipose stromal cells (ASCs). Hypoxia inducible factor-1α (HIF-1α), a key mediator of cellular adaptation to low oxygen levels, is emerging as a novel prognostic marker in breast cancer. We have identified the presence of a consensus HIF-1α binding motif overlapping with the proximal CRE of aromatase PII. However, the regulation of aromatase expression by HIF-1α in breast cancer has not been characterized. This study aimed to characterize the role of HIF-1α in the activation of aromatase PII.

**Methods:**

HIF-1α expression and localization were examined in human breast ASCs using quantitative PCR (QPCR), Western blotting, immunofluorescence and high content screening. QPCR and tritiated water-release assays were performed to assess the effect of HIF-1α on aromatase expression and activity. Reporter assays and chromatin immunoprecipitation (ChIP) were performed to assess the effect of HIF-1α on PII activity and binding. Treatments included PGE_2 _or DMOG ((dimethyloxalglycine), HIF-1α stabilizer). Double immunohistochemistry for HIF-1α and aromatase was performed on tissues obtained from breast cancer and cancer-free patients.

**Results:**

Results indicate that PGE_2 _increases HIF-1α transcript and protein expression, nuclear localization and binding to aromatase PII in human breast ASCs. Results also demonstrate that HIF-1α significantly increases PII activity, and aromatase transcript expression and activity, in the presence of DMOG and/or PGE_2_, and that HIF-1α and CREB1 act co-operatively on PII. There is a significant increase in HIF-1α positive ASCs in breast cancer patients compared to cancer-free women, and a positive association between HIF-1α and aromatase expression.

**Conclusions:**

This study is the first to identify HIF-1α as a modulator of PII-driven aromatase expression in human breast tumor-associated stroma and provides a novel mechanism for estrogen regulation in obesity-related, post-menopausal breast cancer. Together with our on-going studies on the role of AMP-activated protein kinase (AMPK) in the regulation of breast aromatase, this work provides another link between disregulated metabolism and breast cancer.

## Introduction

Epidemiological studies indicate that the proportion of estrogen-dependent breast cancer cases is dramatically increased in postmenopausal women and this, despite low levels of estrogens found in the circulation. Postmenopausally, breast cancer risk also increases with obesity [1]. After menopause, when the ovaries no longer produce measurable amounts of estrogens, an increase in locally produced estrogens within the tumor and surrounding adipose tissue is believed to drive tumor growth via the action of markedly high levels of the aromatase enzyme (reviewed in [2]). The enhanced local expression of aromatase within the breast is mediated via promoter switching from distal promoter I.4 to the alternative proximal promoter II (PII) on the *CYP19A1 *gene in response to inflammatory mediators derived from the tumor, such as prostaglandin E_2 _(PGE_2_) [3,4]. A recent study demonstrated that PGE_2 _is also increased in breast tissues from overweight and obese women and is associated with higher aromatase transcript expression [5]. One of the transcription factors shown to be involved in this process is cAMP response element (CRE) binding protein-1 (CREB1) which binds to the proximal and distal CREs on PII, and stimulates the expression of aromatase [6]. CREB1-coactivators, including CRTC2 [7], CBP [8] and p300 [9], are also known to regulate PII-driven aromatase expression.

Many breast cancers are associated with heterogeneously distributed hypoxic tissue areas within the tumor mass [10] and hypoxia inducible factor-1α (HIF-1α) is found to be a key mediator of hypoxia-mediated tumor responses (reviewed in [11]). Previous studies have demonstrated that HIF-1α is a novel prognostic marker in determining the aggressive phenotype of breast cancer [12,13] and is emerging as a potential target for cancer treatment [14,15]. HIF-1 consists of two subunits, namely HIF1-α and HIF-1β, which belong to the basic-helix-loop-helix (bHLH) protein family containing a per-aryl hydrocarbon receptor nuclear translocator-sim (PAS) domain [16]. HIF-1β is continuously expressed and HIF-1α is continuously synthesized and degraded under normoxic conditions mainly through ubiquitin-proteasome dependent pathways after hydroxylation by prolyl-hydroxylases (PHDs) [17,18]. Under hypoxic conditions, HIF-1 is stabilized and binds to core hypoxia response elements (HREs) containing the 5'-RCGTG-3' sequence [19] with other transcription factors, such as CBP/p300 via its CH1 domain [20], which results in the transcriptional activation of hypoxia-regulated genes including vascular endothelial growth factor (VEGF), known to promote angiogenesis (reviewed in [21]). In PC-3 ML human prostate cancer cells [22] and in HCT116 human colon carcinoma cells [23], it was demonstrated that PGE_2 _and hypoxia act both independently and synergistically to increase HIF-1α protein levels, and further demonstrated the time-dependent nuclear accumulation of HIF-1α in response to PGE_2._

We have identified a putative HRE which overlaps with the proximal CRE of aromatase PII. These findings led us to hypothesize that HIF-1α may have a role in regulating aromatase expression in response to the tumor-derived and obesity-associated factor, PGE_2_, in breast ASCs.

## Materials and methods

### Plasmids

The *CYP19A1 *PII-516 luciferase reporter plasmid, which contains 502 bp (-516 to -14) of the proximal promoter PII was generated as previously described [24]. The HA-HIF-1α-pcDNA vector (Addgene, Cambridge, MA, USA), plasmid 18949) was generated as previously described [25]. The pCMV.CREB1 plasmid was purchased from Promega Australia (Alexandria, NSW, Australia).

### Human tissue, cell culture

Primary human breast ASCs were isolated by collagenase digestion of subcutaneous adipose tissue from women undergoing reduction mammoplasty and cultured in Waymouth's medium (Life Technologies Australia Pty Ltd, Mulgrave, VIC, Australia), as previously described [26]. The studies have been approved by Southern Health Human Research Ethics Committee B (#B00109). MCF-7 cells (human breast adenocarcinoma cell line) were cultured in Dulbecco's modified Eagle's medium (Invitrogen, USA). Before treatments, cells were serum-starved for 24 hours in medium containing 0.1% bovine serum albumin. Treatments included prostaglandin E_2 _(PGE_2_, 1 μM) and dimethyloxalglycine (DMOG, 100 μM; prolyl-4-hydroxylase inhibitor which stabilizes HIF-1α) purchased from Sigma-Aldrich Pty Ltd (Sydney, NSW, Australia). Sections of formalin-fixed and paraffin-embedded breast tissues from 10 Japanese breast cancer patients (IDC, invasive ductal carcinoma and DCIS, ductal carcinoma *in situ*), with differing hormone receptor status and grade (Additional file 1, Table S1), and 10 cancer-free women were used for double immunohistochemistry studies. Japanese female patients with IDC and DCIS were obtained from St. Luke's International Hospital (Tokyo, Japan). The informed consent being obtained from these patients before surgery and the research protocols were approved by the ethics committee at St Luke's International Hospital (2010-509). All the clinical data were retrieved from the relevant patient's file and the histological grade was independently evaluated.

### Nuclear extraction and Western blot analysis

Primary breast ASCs were cultured on 10 cm plates and treated as described above. Nuclear extracts were obtained as described in the Abcam technical website with minor modifications, including addition of cobalt chloride (1 mM) and protease inhibitor cocktail tablet-complete mini from Roche Products Pty Ltd (Dee Why, NSW, Australia). BCA protein assay (Thermo Fisher Scientific Australia Pty Ltd, Scoresby, VIC, Australia) was performed to quantify protein amount according to the manufacturer's instructions. A total of 10 μg of nuclear protein diluted in loading buffer containing β-mercaptoethanol was run on 10% denaturing polyacrylamide gel and transferred to nitrocellulose membrane. HIF-1α and histone H3 protein levels were detected using HIF-1α (sc-10790; 1/200 dilution, Santa Cruz Biotechnology, Santa Cruz, CA, USA), histone H3 (ab1791; 1/10,000 dilution; Sapphire Bioscience Pty Ltd, Waterloo, NSW, Australia) and Alexa Fluor 700 goat anti-rabbit secondary (1/10,000 dilution, Invitrogen, USA) antibodies using the Odyssey infrared imaging system (LI-COR Biosciences, Lincoln, NE, USA). The intensity of the bands detected from Western blotting was quantified using densitometric analysis.

### Reverse transcription and quantitative PCR (QPCR)

Total RNA was extracted using the RNeasy Mini Kit (QIAGEN Pty Ltd, Chadstone, VIC, Australia) and 0.3 to 1 μg of RNA was reverse transcribed using the AMV RT Kit using oligo-dT primer (Promega Australia, Alexandria, NSW, Australia) as directed by the manufacturer. DNA was digested using the DNA-free DNase Treatment and Removal Kit ((Life Technologies Australia Pty Ltd, Mulgrave, VIC, Australia). QPCR was performed on the LightCycler using LightCycler FastStart DNA Master SYBR Green l kit (Roche Products Pty Ltd, Dee Why, NSW, Australia). Quantification of human HIF-1α, human aromatase and β-actin or 18 s (housekeeping genes) transcripts was carried out using primers hHIF-1α F: 5'-GTACCCTAACTAGCCGAGGAAGAA-3', hHIF-1α R: 5'-GTGAATGTGGCCTGTGCAGT-3', hArom F: 5'-TTGGAAATGCTGAACCCGAT-3', hArom R: 5'-CAGGAATCTGCCGTGGGGAT-3', β-actin F: 5'-TGCGTGACATTAAGGAGAAG-3', β-actin R: 5'- GCTCGTAGCTCTTCTCCA -3', 18S-F: 5'-CGGCTACCACATCCAAGGAA-3' and 18S-R: 5'GCTGGAATTACCGCGGCT-3'. Cycling conditions were one cycle at 95^°^C for 10 minutes followed by 40 cycles of 95^°^C for 10 sec, 59^°^C for 6 sec and 72^°^C for 4 sec for HIF-1α, 40 cycles of 95^°^C for 10 sec, 60^°^C for 5 sec and 72^°^C for 10 sec for aromatase and 30 cycles of 95^°^C for 10 sec, 59^°^C for 5 sec and 72^°^C for 10 sec for β-actin or 18 s. All the samples were quantified using standards of known concentrations and corrected for abundance with the housekeeping gene β-actin or 18 s.

### Immunofluorescence and confocal imaging

HIF-1α protein was visualized in primary breast ASCs using immunofluorescence and confocal microscopy. ASCs were plated onto coverslips and cultured until they reached approximately 70% confluency. Cells were serum-starved overnight and treated for 24 hrs. Immunofluorescence was performed as previously described [27], using HIF-1α antibody (sc-10790; 1/2,000 dilution, Santa Cruz Biotechnology, USA) and lamin B1+B2 antibody (1/1,000 dilution, Abcam, USA) for nuclear stain and visualized using alexa fluor-546 (red) and -488 (green) from Invitrogen (USA), respectively, using confocal microscopy (Olympus Australia Pty Ltd, Mt Waverley, VIC, Australia).

### High content screening

Primary breast ASCs cells were plated in 96-well plates at a density of 6,000 cells per well. One day after plating, the cells were serum-starved for 24 hours and treated with PGE_2 _for 6 hours. Fixation was carried out in ice-cold methanol for 40 minutes at -20^°^C, followed by 2 × PBS washes. Cells were blocked in 0.5% BSA/PBS for 30 minutes and incubated with HIF-1α antibody (sc-10790; 1/2,000 dilution in 0.5% BSA/PBS, Santa Cruz Biotechnology, USA) overnight at 4^°^C with gentle rocking. After 2 × PBS washes, alexa fluor 488 goat anti-rabbit secondary antibody (1/1,000 dilution, Invitrogen, USA) and Hoechst nuclear counterstain (1/5,000 dilution) in 0.5% BSA/PBS were applied for 90 minutes. In order to quantitate nuclear fluorescence, images were captured on an ArrayScan VTI instrument (Thermo Fisher Scientific Australia Pty Ltd, Scoresby, VIC, Australia) and analyzed using Cellomics software (Thermo Fisher Scientific Australia Pty Ltd, Scoresby, VIC, Australia) and the Compartmental Analysis Bioapplication (Thermo Fisher Scientific Australia Pty Ltd). The analysis algorithm used Hoechst fluorescence to define a mask used to measure nuclear fluorescence. Threshold for nuclear staining was determined by assessing nuclear intensity in negative control samples and set to two standard deviations. Cells with nuclear HIF-1α pixel intensity ≥ 230 were considered HIF-1α positive.

### Chromatin immunoprecipitation (ChIP)

Primary breast ASCs treated with PGE_2 _or DMOG for 45 minutes were used for ChIP to examine endogenous binding of HIF-1α to aromatase PII using the ChIP-IT express kit (Active Motif, Carlsbad, CA, USA) as directed by the manufacturer with minor modifications [7]. Briefly, cells were fixed using 1% formaldehyde to cross-link and preserve endogenous protein-DNA interactions. The DNA was then sheared into small fragments using sonication at 20% amplitude, seven times for 30 sec pulses. Specific protein-DNA complexes were immune-precipitated using HIF-1α antibody (Santa Cruz Biotechnology, USA), IgG or water as controls. QPCR was then performed using primers flanking the CREs of *CYP19A1 *PII (PII-ChIP-F: 5'-TTTCCACACTACCGTTGGCCG-3' and PII-ChIP-R: 5'-GGCAATCTTCTTCCCTTGAAGC-3'), and normalized to input DNA [7].

### Reporter gene assays

MCF-7 cells were transfected with wild type or proximal CRE mutated *CYP19A1 *PII-516 luciferase constructs, with/without human HIF-1α-pcDNA (Addgene, Cambridge, MA, USA) and/or human CREB1-pcDNA or with HIF-1α siRNA (sc-35561, Santa Cruz Biotechnology, USA) or control siRNA-A (sc-37007, Santa Cruz Biotechnology, USA), using the cell line nucleofector kit V, program E-014 (Lonza, Tullamarine, VIC, Australia), according to the manufacturer's instructions. β-galactosidase was co-transfected and used as a transfection control. After transfection, cells were serum-starved and treated with FSK/PMA and/or DMOG for 24 hours. Luciferase reporter assays were carried out using the Dual-Glo Luciferase Assay System (Promega, USA) according to the manufacturer's protocol and data were normalized to β-galactosidase activity.

### Aromatase activity assay (tritiated water-release assay)

Primary breast ASCs and MCF-7 cells were plated in six-well plates. MCF-7 cells were transfected with 2 μg of human HIF-1α-pcDNA (Addgene, USA) or 3 μl of HIF-1α siRNA (sc-35561, Santa Cruz Biotechnology, USA) or control siRNA-A (sc-37007, Santa Cruz Biotechnology, USA) using lipofectamine transfection reagent (Invitrogen, USA), as directed by the manufacturer. ASCs and MCF-7 cells were serum-starved for 24 hours and treated with specified reagents. Aromatase activity in these cells was measured using the tritiated water-release assay using androst-4-ene-3, 17-dione (NET926001MC, PerkinElmer, Glen Waverley, VIC, Austalia) as a substrate, as previously described [28]. Specific activity was normalized to total protein amount.

### Double immunohistochemistry (IHC)

Formalin-fixed paraffin-embedded tissue sections from breast cancer and cancer-free patients were de-waxed in xylene and were rehydrated through descending concentrations of ethanol solutions to distilled H_2_O. Tissue sections were incubated in 10% horse serum in CAS-block (Invitrogen, USA) for 30 minutes. Previously characterized aromatase mouse monoclonal primary antibody 677 (1/250 dilution from 2.6 mg/ml stock in 0.5% BSA/PBS, [29]) was added to the slides and incubated overnight at 4°C. After washing in PBS, biotinylated universal secondary antibody (1/200 dilution, Vectastain Universal ABC-AP kit, Abacus ALS, East Brisbane, QLD, Australia) was applied for 30 minutes and then incubated with Vectastain ABC-AP reagent for 30 minutes. Alkaline phosphatase substrate (Vector blue alkaline phosphate substrate kit, Vector Laboratories, USA) was added until the desired cytoplasmic blue stain intensity developed and the reaction stopped with distilled H_2_O.

Slides were then subjected to antigen retrieval in an autoclave, at 121^°^C for 5 minutes in Tris EDTA pH 9 (10 mM Tris base, 1 mM EDTA). After cooling for 30 minutes, sections were washed in PBS and incubated in 10% horse serum in CAS-block (Invitrogen, USA) for 30 minutes. The HIF-1α primary antibody (sc-10790; 1/250 dilution in 0.5% BSA/PBS, Santa Cruz Biotechnology, USA) was added to the slides and incubated overnight at 4^°^C. After washing in PBS, endogenous peroxidase activity was blocked with 0.3% hydrogen peroxide for 30 minutes. Anti-rabbit IgG secondary antibody (1/1,000 dilution, Vectastain ABC-AP kit-rabbit IgG, Vector laboratories, USA) was applied for 30 minutes and then incubated with Vectastain ABC-AP reagent for 30 minutes. Slides were then stained with 3,3'-diaminobenzidine tetrahydrochloride (DAB, Sigma-Aldrich, USA) until desired nuclear brown stain intensity developed and the reaction was stopped with distilled H_2_O. Finally, the sections were mounted with fluorsave reagent (Merck Pty Ltd, Kilsyth, VIC, Australia).

Double immunohistochemically stained slides for HIF-1α and aromatase were evaluated independently by two observers (NUS and MS or NUS and KAB). The ASCs were examined using systematic random sampling on a stereology microscope (Olympus Australia Pty Ltd, Mt Waverley, VIC, Australia) with the aid of CAST-Grid version 1.60 (Olympus Australia Pty Ltd, Mt Waverley, VIC, Australia) and categorized into four groups including HIF-1α positive and aromatase positive, HIF-1α positive and aromatase negative, HIF-1α negative and aromatase positive, and HIF-1α negative and aromatase negative (Additional file 2, Figure S1).

### Statistical analysis

All data were expressed as mean ± standard error (SE). Two-tailed Student's *t *test was performed for experiments comparing two groups. For experiments with multiple comparisons, statistical analysis was done using one-way ANOVA followed by Bonferroni's multiple comparison test. Statistical significance was defined as *, *P *< 0.05; **, *P *≤ 0.005; ***, *P *≤ 0.0005, ****, *P *≤ 0.0001. Data analysis was performed using GraphPad Prism version 5.00 (GraphPad Software, San Diego, CA, USA).

## Results

### PGE_2 _increases HIF-1α expression and nuclear localization in primary human breast ASCs

**Figure 1:**
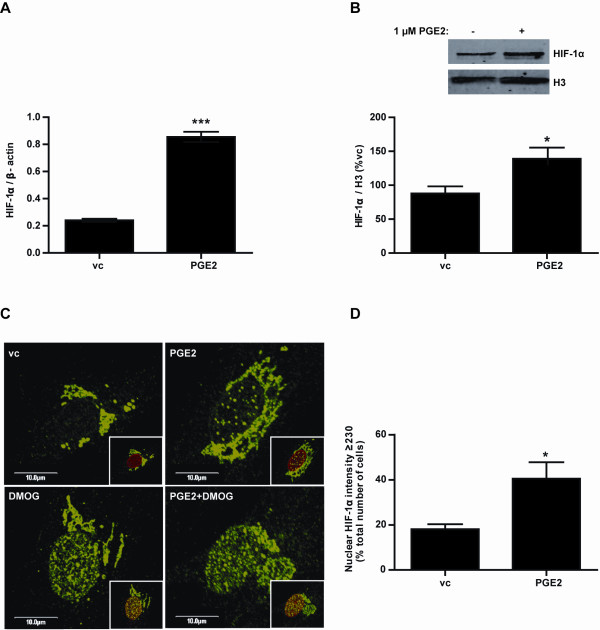
**Effect of PGE_2 _on HIF-1α expression and nuclear localization in primary human breast ASCs**. PGE_2 _caused a significant increase in HIF-1α transcript (**A**) and nuclear protein expression (**B**). Confocal microscopy demonstrated that HIF-1α (green) is mainly perinuclear in breast ASCs under basal conditions (**C**, top left) and that PGE_2 _stimulates the translocation of HIF-1α to the nucleus (C, top right). Treatment with DMOG (C, bottom left) and DMOG with PGE2 (C, bottom right) caused a much higher HIF-1α staining in the nucleus. The merged lamin B1+B2 nuclear stain (red) and HIF-1α are found as insets at the bottom right of each image. The percentage of cells positive for nuclear HIF-1α was also shown to be significantly increased with PGE_2 _treatment (**D**). vc = vehicle control, *n *= 3, repeated twice. Confocal images are representative of the majority of cells examined.

The effect of PGE_2 _on HIF-1α transcript and protein expression was examined in primary breast ASCs. HIF-1α mRNA was significantly increased with PGE_2 _treatment (Figure 1A). Moreover, Western blotting demonstrated that PGE_2 _also caused a significant increase in HIF-1α protein abundance in the nucleus (Figure 1B). The subcellular localization of endogenously expressed HIF-1α was examined using immunofluorescence and confocal microscopy after treating with PGE_2 _and/or DMOG. Results demonstrated that punctate staining for HIF-1α appears in the nucleus after PGE_2 _treatment (Figure 1C, top right) compared to cytoplasmic localization under basal conditions (Figure 1C, top left). Furthermore, DMOG (Figure 1C, bottom left) and DMOG with PGE_2 _(Figure 1C, bottom right) resulted in more intense staining for HIF-1α in the nucleus. Results from high content screening also demonstrated that the percentage cells positive for HIF-1α in the nucleus (Figure 1D) was significantly increased in response to PGE_2 _treatment.

### HIF-1α binds to aromatase promoter PII and increases aromatase expression and activity in primary breast ASCs

**Figure 2:**
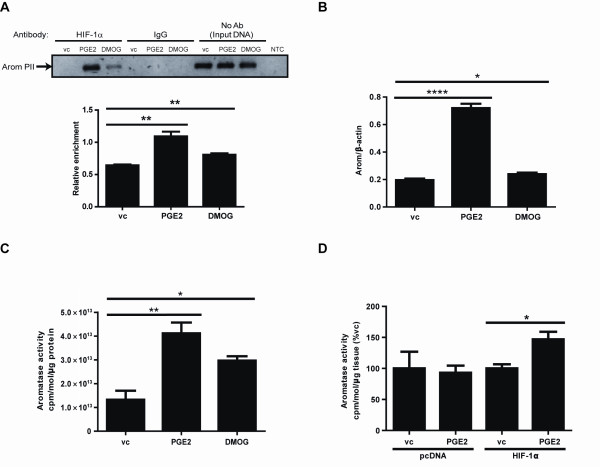
**Role of HIF-1α in aromatase regulation**. (**A**) ChIP demonstrated that PGE_2 _and DMOG significantly stimulate the endogenous binding of HIF-1α to aromatase PII. Treatment of ASCs with PGE_2 _or DMOG caused a significant increase in aromatase transcript expression (**B**) and activity (**C**). (**D**) HIF-1α overexpression in MCF-7 cells significantly increased aromatase activity in the presence of PGE_2_. vc = vehicle control, *n *= 3, repeated twice.

The endogenous binding of HIF-1α to aromatase PII was evaluated by ChIP. Treatment with either PGE_2 _or DMOG resulted in a significant increase in binding of HIF-1α to aromatase PII compared to vehicle control (Figure 2A). Consistent with these findings, treatment of primary human breast ASCs with PGE_2 _or DMOG also caused a significant increase in aromatase transcript expression (Figure 2B) and aromatase activity (Figure 2C). Overexpression of HIF-1α in MCF-7 cells treated with PGE_2 _also resulted in increased aromatase activity (Figure 2D).

### HIF-1α acts cooperatively with CREB1 to increase PII activity breast ASCs

**Figure 3:**
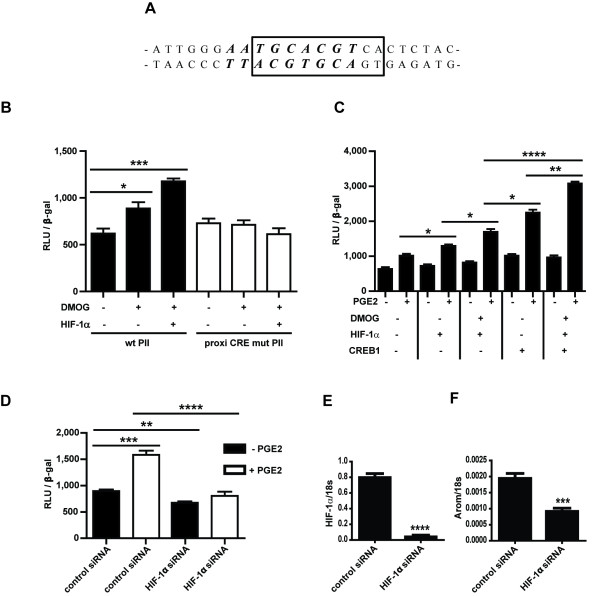
**HIF-1α is necessary and acts cooperatively with CREB1 to induce PII activity in response to PGE_2_**. (**A**) A putative HRE (italic) was found to overlap with the proximal CRE (boxed) of aromatase PII. (**B**) Reporter assays demonstrated that mutation of the proximal CRE of aromatase PII inhibited the HIF-1α/DMOG-mediated effect on PII activity. (**C**) PII activity was significantly increased in HIF-1α transfected cells, treated with DMOG and PGE_2_, and co-transfection with CREB1 resulted in a further increase in PII activity compared to cells transfected with either HIF-1α or CREB1 alone. Knockdown of HIF-1α (**E**) using siRNA significantly reduced PII activity (**D**) and aromatase transcript expression (**F**) and suppressed the PGE_2_-mediated effect on PII activity (D). β-gal, β-galactosidase activity; RLU, relative luciferase units. *N *= 3, all experiments repeated twice.

PROMO 3.0 online putative transcription factor binding site predictor [30] was used to identify a putative binding site of HIF-1α on aromatase PII [31,32]. Results demonstrated that the predicted HRE overlaps with the proximal CRE of aromatase PII (Figure 3A).

To determine the effect of HIF-1α on aromatase PII activity, luciferase reporter assays were performed in MCF-7 cells, transfected with HIF-1α and wild type or proximal CRE mutated *CYP19A1 *PII-516 luciferase reporter constructs and treated with DMOG. Cells transfected with HIF-1α treated with DMOG and DMOG alone showed a significant increase in promoter II activity, and this HIF-1α/DMOG-mediated effect on promoter II activity was completely abolished using a proximal CRE-mutated PII reporter construct (Figure 3B). As the putative HRE overlaps with the proximal CRE on aromatase PII, experiments were performed to determine whether HIF-1α acted cooperatively or competitively with CREB1. MCF-7 cells were transfected with HIF-1α and/or CREB1 together with the *CYP19A1 *PII-516 luciferase reporter construct, and then treated with DMOG and/or PGE_2_. PII activity was significantly increased in HIF-1α transfected cells in the presence of PGE_2 _or DMOG, and treatment with both PGE_2 _and DMOG led to a further significant increase in PII activity (Figure 3C). Interestingly, cells transfected with both HIF-1α and CREB1 showed a further increase in PII activity (*P *≤ 0.005) with both PGE_2 _and DMOG compared to cells transfected with either HIF-1α or CREB1 alone (Figure 3C). Hence, HIF-1α and CREB1 act cooperatively to increase PII activity.

### HIF-1α is necessary for the PGE_2_-mediated induction of aromatase PII

Reporter assays were also performed to examine the requirement of HIF-1α for the PGE_2_-mediated increase in PII activity. MCF-7 cells transfected with HIF-1α siRNA showed a significant reduction in aromatase PII activity (Figure 3D) and aromatase transcript (Figure 3F), and the PGE_2_-mediated effect on PII via HIF-1α was suppressed (Figure 3D).

### HIF-1α is increased in ASCs from tumor-bearing breast tissues compared to cancer free breast tissue

**Figure 4:**
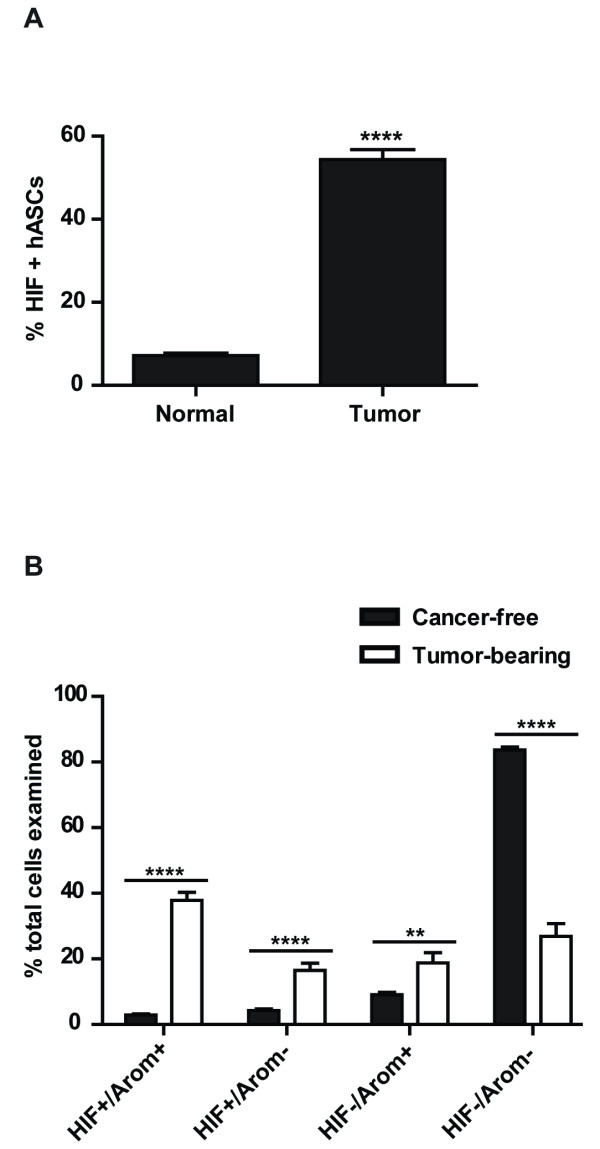
**HIF-1α and aromatase expression in ASCs from tumor-bearing breast tissue compared to cancer-free using immunohistochemistry**. (**A**) Percentage of HIF-1α positive ASCs from tumor-bearing breast tissue was significantly increased compared to tissue from cancer-free women. (**B**) Double-positive and single-positive ASCs for HIF-1α and aromatase were shown to be significantly increased in tumor-bearing compared to cancer-free tissues. Double negative ASCs for HIF-1α and aromatase were significantly reduced in tumor-bearing compared to cancer-free tissues. *n *= 10 for tumor-bearing breast tissue; *n *= 10/cancer-free breast tissue.

Double immunohistochemistry was performed on formalin-fixed, paraffin-embedded (FFPE) tissues from breast cancer patients and cancer-free women. Results demonstrated that there is a significant increase in the percentage of HIF-1α positive ASCs in breast cancer patients compared to cancer-free women (Figure 4A). Interestingly, ASCs, which were HIF-1α and aromatase double-positive and which were single-positive for HIF-1α or aromatase, were significantly increased in tumor patients compared to cancer-free women (Figure 4B). Furthermore, double negative ASCs were significantly decreased in tumor cases compared to normal (Figure 4B), and the percentage of cells either double-positive or double-negative was significantly higher than single-positive cells (analysis not shown), suggesting a positive association between HIF-1α and aromatase expression.

## Discussion

**Figure 5:**
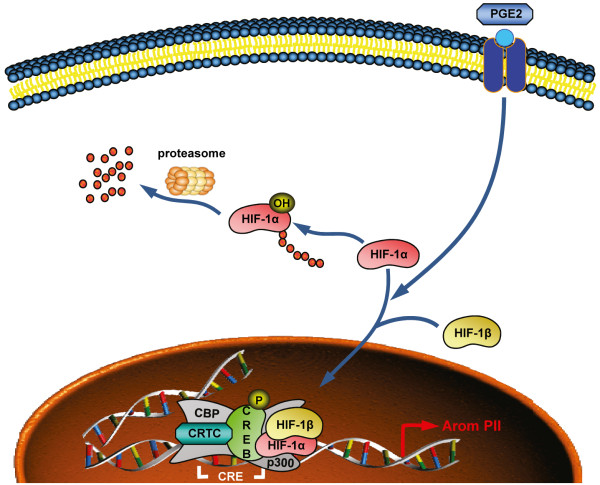
**Model of the PGE_2_-mediated regulation of aromatase expression by HIF-1α in breast ASCs**. Tumor-derived factor PGE_2 _increases HIF-1α transcript expression and nuclear localization. HIF-1α dimerizes with HIF-1β and then translocates to nucleus where it interacts with the proximal CRE of aromatase PII. HIF-1α together with CREB, CRTCs, CBP and p300 act to increase the PII-driven expression of aromatase.

In this study, novel evidence is provided for the regulation of HIF-1α and its role in regulating aromatase expression in adipose stromal cells in the context of obesity and breast cancer. Namely, that HIF-1α expression and nuclear localization are increased in response to PGE_2 _and stimulate the promoter II-driven expression of aromatase in breast ASCs via binding to the proximal CRE (Figure 5).

HIF-1α has been shown to be overexpressed in many different types of tumors including those of the ovary, prostate and breast [33]. However, the expression and regulation of HIF-1α in cancer-associated adipose stromal cells is less well characterized. Here, we demonstrate that HIF-1α transcript, protein expression and protein nuclear localization are increased in breast ASCs in response to the tumor-derived factor PGE_2_. Consistent with these observations, we also demonstrate that in clinical samples, the number of HIF-1α positive ASCs is increased in breast cancer patient samples compared to cancer-free breast tissue. Despite the limited number of patient samples examined and these being from women with differing tumor grade, the dramatic increase observed was highly conserved among all patients. However, the majority of cases were postmenopausal women with hormone receptor positive tumors. Nevertheless, it has been reported that the majority of breast tumors overexpress COX-2 and secrete high levels of PGE_2 _[34,35]. Our results are consistent with observations demonstrating that PGE_2 _causes the stabilization of HIF-1α independent of hypoxia in PC-3 ML human prostate cancer cells [22], HCT116 human colon carcinoma cells [23] and AGS gastric carcinoma cells [36]. However, to our knowledge, this is the first report to demonstrate an increase in HIF-1α transcript levels in response to PGE_2 _in ASCs.

The PGE_2_-induced up-regulation of HIF-1α has been shown to be mediated through EP2 and EP4 receptor activation in PC-3 ML cells [22] and the EP1 receptor alone in HCT116 cells [23]. In human embryonic kidney cells expressing the human EP1 receptor, PGE_2 _has been shown to up-regulate HIF-1α protein expression in a time-dependent manner under normoxic conditions [37]. Interestingly, the expression of EP receptors has been demonstrated in breast ASCs and the regulation of aromatase by PGE_2 _in these cells has been shown to be dependent on activation the EP1 and EP2 receptors [4,38]. These data suggest that aromatase up-regulation via EP receptor activation may involve the induction of HIF-1α. Indeed, HIF-1α increases the activity of aromatase PII, and the increased nuclear localization and punctuate appearance of HIF-1α in response to PGE_2 _is also associated with the increased binding of HIF-1α to aromatase PII via the proximal CRE. The regulation of aromatase by HIF-1α has also recently been examined in the context of placental aromatase regulation. In that case, aromatase expression is mediated by distal placental-specific promoter I.1 and is dependent on binding of estrogen-related receptor γ (ERRγ) to the promoter [39]. Hypoxia has been shown to cause the HIF-1α-dependent down-regulation of ERRγ [39]. Contrary to our findings, the HIF-1α-mediated effects on PI.1 are inhibitory and appear to be indirect.

Promoter analysis revealed that a putative core HRE sequence is present on the antisense strand of aromatase PII and overlaps with the proximal CRE, suggesting that HIF-1α may interact with CREB1 in ASCs. Previous studies have demonstrated that CREB1 binds to the HRE in the plasminogen activator inhibitor-1 (PAI-1) promoter [40] and HIF-1α can also interact with ATF2/CBP/p300 [41-43]. There is a significant elevation in CREB1 transcript expression in breast tumor tissues compared to non-neoplastic breast tissues and this is positively associated with poor prognosis, metastatic disease and nodal involvement [44,45]. Interestingly, the present study demonstrates that HIF-1α alone can stimulate aromatase promoter PII and that it also acts cooperatively with CREB1 to increase aromatase PII activity.

The local biosynthesis of estrogens from breast ASCs is considered a key mediator of tumor cell growth in postmenopausal breast cancer, and increased PII activity accounts for the majority of transcripts detected [3]. We have observed that the majority of ASCs in the tumor-bearing tissue are either double-positive or double-negative for HIF-1α and aromatase expression, suggesting an association between the two proteins. These findings support our *in vitro *data demonstrating that HIF-1α directly stimulates aromatase expression.

Collectively, the results obtained in this study show for the first time that HIF-1α activates PII-driven aromatase expression in breast ASCs in response to PGE_2_, independent of oxygen availability. Hence, this specific association is likely to be an important mechanism for the regulation of estrogen biosynthesis in obesity-related, postmenopausal breast cancer. Third-generation aromatase inhibitors (AIs) are currently the most effective treatment and have been shown to be superior to tamoxifen for hormone receptor positive, postmenopausal breast cancer as aromatase catalyses the conversion of circulating androgenic precursors to estrogens [46-48]. However, many women cease use of AIs due to increasingly severe side-effects associated with their use [49-51]. Currently, small molecule inhibitors of HIF-1α are being tested in the clinical setting (reviewed in [52]). We believe that better understanding of the regulation of aromatase PII will allow us to target aromatase expression specifically within the breast, leaving sites such as the bone, brain and heart, where estrogens have beneficial effects, unaffected.

## Conclusions

This study demonstrates that HIF-1α, a master regulator of oxygen homeostasis, stimulates PII-driven aromatase expression in human breast ASCs with other transcription factors, including CREB1, in response to tumor-derived and obesity-associated inflammatory mediator PGE_2_. Our findings of HIF-1α in tumor-associated breast stroma implicate its potential as a therapeutic target in obesity-related, postmenopausal breast cancer.

## Abbreviations

AIs: aromatase inhibitors; AMPK: AMP-activated protein kinase; ASC: adipose stromal cells; bHLH: basic-helix-loop-helix; ChIP: chromatin immunoprecipitation; CREB1: CRE binding protein 1; CREs: cAMP response elements; DCIS: ductal carcinoma *in situ; *DMOG: dimethyloxalglycine; ERRγ: estrogen-related receptor γ; FFPE: formalin-fixed, paraffin-embedded; HIF-1α: hypoxia inducible factor 1 alpha; HREs: hypoxia response elements; IDC: invasive ductal carcinoma; PAI-1: plasminogen activator inhibitor-1; PAS: per-aryl hydrocarbon receptor nuclear translocator-sim; PBS: phosphate-buffered saline; PGE_2_: Prostaglandin E_2_; PHDs: prolyl-hydroxylases; PII: promoter II; QPCR: quantitative PCR; SE: standard error; VEGF: vascular endothelial growth factor.

## Competing interests

The authors declare that they have no competing interests.

## Authors' contributions

NUS and KAB designed all the experiments. NUS conducted all the experiments with help from FY, MMD, MS, KMM, HS and SBF. The paper was written by NUS and KAB, ERS, SBF and HS were involved in manuscript revision. KAB and ERS contributed to the conception of the project. All authors revised the manuscript and gave their final approval.

## Supplementary Material

Additional file 1 **Table S1: Clinicopathological data of breast cancer patients**. Formalin-fixed and paraffin-embedded breast tissues from 10 Japanese breast cancer patients (IDC and DCIS), with differing hormone receptor status and grade used in Figure 4 are listed. Click here for file

Additional file 2 **Figure S1: Classification of ASCs for double immunohistochemistry**. (**A, B**; arrows) HIF-1α and aromatase double positive cells. (**C, D**; arrows) HIF-1α positive and aromatase negative cells. (**E, F**; filled arrows) HIF-1α negative and aromatase positive cells. (E, empty arrow) HIF-1α positive and aromatase positive cell. (F, empty arrow) HIF-1α and aromatase double negative cell. Blue color in the cytoplasm as a result of Vector blue colorimetric reaction represents aromatase immunoreactivity while brown color in the nuclei as a result of DAB colorimetric reaction represents HIF-1α immunoreactivity. Click here for file
