# Supplementary material for: HIF-1α stimulates aromatase expression driven by prostaglandin E2 in breast adipose stroma
Source: Breast Cancer Res. 2013 Apr 8;15(2):R30. doi: 10.1186/bcr3410 (PMC3672802; doi:10.1186/bcr3410)
Supplement: Additional file 1 — Table S1: Clinicopathological data of breast cancer patients. Formalin-fixed and paraffin-embedded breast tissues from 10 Japanese breast cancer patients (IDC and DCIS), with differing hormone receptor status and grade used in Figure 4 are listed. [file bcr3410-S1.DOC]

|  | **Age** | **Tumor type** | **Nuclear grade** | **Nottingham's histological grade** | **ER**  **(Allred score)** | **PgR**  **(Allred score)** | **HER2**  **(Allred score)** |
| --- | --- | --- | --- | --- | --- | --- | --- |
| **1** | 69 | DCIS | II | N/A | 8 | 6 | 1 |
| **2** | 61 | IDC, papillotubular carcinoma | I | N/A | 8 | 8 | 0 |
| **3** | 36 | IDC, solid-tubular carcinoma | III | N/A | 0 | 0 | 1 |
| **4** | 75 | IDC, solid-tubular carcinoma | III | N/A | 8 | 5 | 1 |
| **5** | 66 | DCIS | I | N/A | 8 | 7 | 1 |
| **6** | 57 | IDC, scirrhous carcinoma * | III | III | 8 | 7 | 2 |
| **7** | 76 | IDC, scirrhous carcinoma | I | II | 8 | 7 | 0 |
| **8** | 55 | IDC, scirrhous carcinoma * | I | II | 8 | 3 | 1 |
| **9** | 60 | IDC, scirrhous carcinoma * | II | II | 8 | 7 | 0 |
| **10** | 59 | IDC, scirrhous carcinoma * | III | III | 4 | 0 | 3 |

DCIS: ductal carcinoma in situ; IDC: invasive ductal carcinoma; * post-chemotherapy; N/A: not available
