# Supplementary figures and images for: HIF-1α stimulates aromatase expression driven by prostaglandin E2 in breast adipose stroma
Source: Breast Cancer Res. 2013 Apr 8;15(2):R30. doi: 10.1186/bcr3410 (PMC3672802; doi:10.1186/bcr3410)

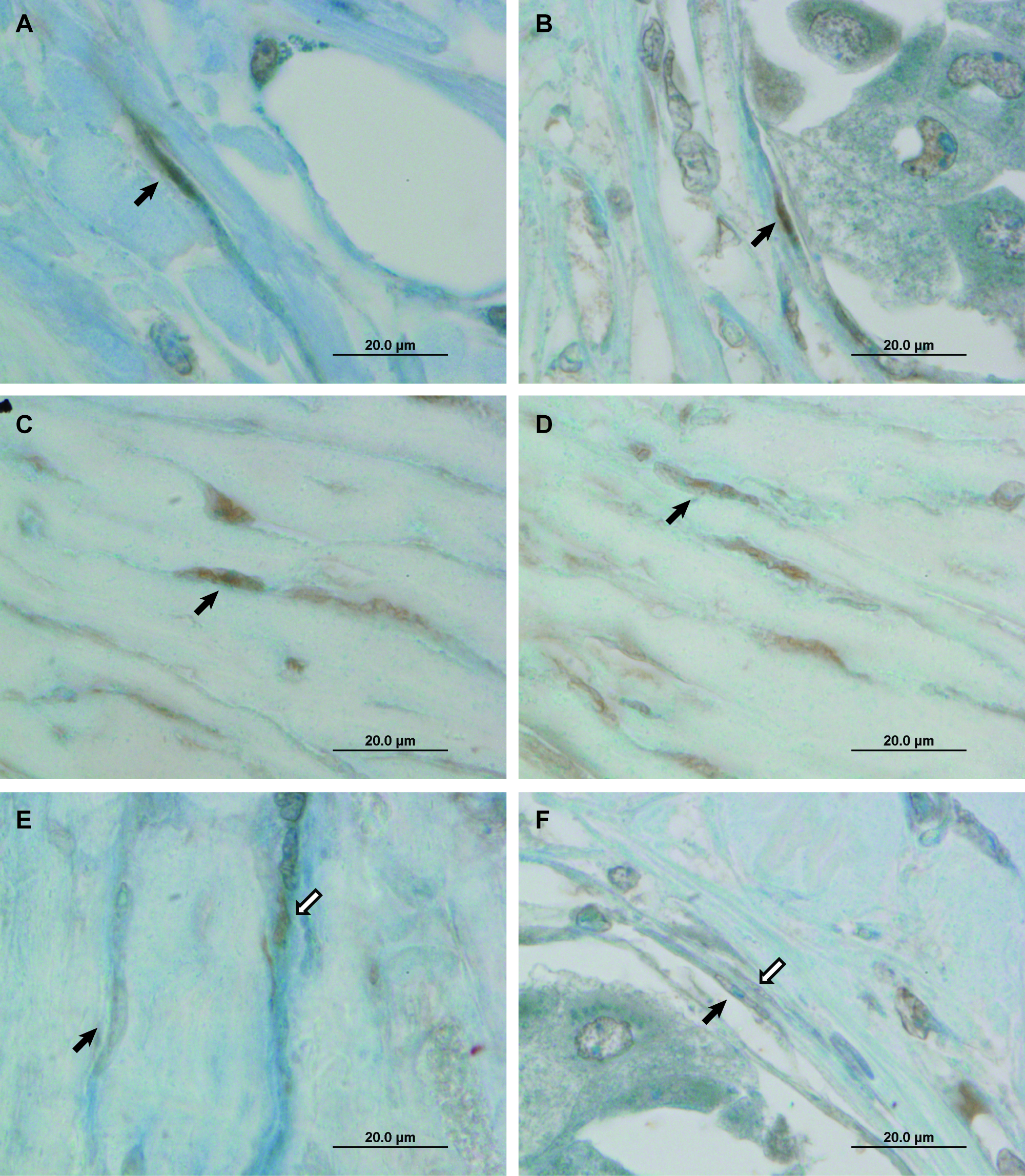

Supplement: Additional file 2 — Figure S1: Classification of ASCs for double immunohistochemistry. (A, B; arrows) HIF-1α and aromatase double positive cells. (C, D; arrows) HIF-1α positive and aromatase negative cells. (E, F; filled arrows) HIF-1α negative and aromatase positive cells. (E, empty arrow) HIF-1α positive and aromatase positive cell. (F, empty arrow) HIF-1α and aromatase double negative cell. Blue color in the cytoplasm as a result of Vector blue colorimetric reaction represents aromatase immunoreactivity while brown color in the nuclei as a result of DAB colorimetric reaction represents HIF-1α immunoreactivity. [file bcr3410-S2.TIFF]
